# Identification of superior parental lines for biparental crossing via genomic prediction

**DOI:** 10.1371/journal.pone.0243159

**Published:** 2020-12-03

**Authors:** Ping-Yuan Chung, Chen-Tuo Liao

**Affiliations:** Department of Agronomy, National Taiwan University, Taipei, Taiwan; USDA-ARS Southern Regional Research Center, UNITED STATES

## Abstract

A parental selection approach based on genomic prediction has been developed to help plant breeders identify a set of superior parental lines from a candidate population before conducting field trials. A classical parental selection approach based on genomic prediction usually involves truncation selection, i.e., selecting the top fraction of accessions on the basis of their genomic estimated breeding values (GEBVs). However, truncation selection inevitably results in the loss of genomic diversity during the breeding process. To preserve genomic diversity, the selection of closely related accessions should be avoided during parental selection. We thus propose a new index to quantify the genomic diversity for a set of candidate accessions, and analyze two real rice (*Oryza sativa* L.) genome datasets to compare several selection strategies. Our results showed that the pure truncation selection strategy produced the best starting breeding value but the least genomic diversity in the base population, leading to less genetic gain. On the other hand, strategies that considered only genomic diversity resulted in greater genomic diversity but less favorable starting breeding values, leading to more genetic gain but unsatisfactorily performing recombination inbred lines (RILs) in progeny populations. Among all strategies investigated in this study, compromised strategies, which considered both GEBVs and genomic diversity, produced the best or second-best performing RILs mainly because these strategies balance the starting breeding value with the maintenance of genomic diversity.

## Introduction

Biparental crossing is a common scheme used for pure-line breeding in self-pollinated crops such as rice (*Oryza sativa* L.), wheat (*Triticum aestivum* L.), soybean (*Glycine max* [L.] Merr.), and oat (*Avena sativa* L.). Plant breeders cross two inbred parental lines to produce the F_1_ population. Then, a subset of diverse individuals from the F_2_ population is selected to produce potential recombination inbred lines (RILs) after several generations of selfing. Parental lines play a fundamental role in line development, and significantly affect the performance of the resulting RILs. However, the identification of superior parental lines from germplasm collections for maximizing selection response in subsequent cycles remains challenging for plant breeders [1, 2]. Another practical concern is that the number of possible crosses in such a breeding program is often far greater than what can be handled in the field. Therefore, developing a method that can identify a limited number of superior parents before field trials would be of great help to plant breeders.

Genomic selection, based on the statistical method of genomic prediction (GP), has been used to improve breeding efficiency in dairy cattle [3] and a variety of crops [4–8]. The main concept of GP is to capture all the effects of quantitative trait loci (QTLs) using high-density DNA markers over the whole genome [9]. The most commonly used DNA markers are single nucleotide polymorphisms (SNPs). A GP model is first built using the phenotypic and genotypic data of a training population. Then, genomic estimated breeding values (GEBVs) for the candidate individuals with known genotypic data are predicted through the resulting GP model. Two kinds of mixed linear model methods are widely employed to obtain GEBVs: (i) best linear unbiased prediction (BLUP) based on markers, and (ii) BLUP based on a genomic relationship matrix. To perform marker-based BLUP, the marker effects are treated as random effects, and GEBVs of individuals are calculated by multiplying their marker scores by these BLUP estimates; the ridge regression BLUP (rr-BLUP) method [9, 10] follows this approach. To perform genomic relationship matrix-based BLUP, the genotypic values of individuals are treated as random effects and estimated through a genomic relationship matrix; this approach is used in the genomic BLUP (GBLUP) model [11, 12].

Gaynor et al. [13] proposed a two-part strategy for implementing genomic selection for line development, which addresses two components: (i) a product development component, to identify inbred lines either for hybrid parent development or cultivar release, and (ii) a population improvement component, to increase the frequency of favorable alleles through rapid recurrent genomic selection. Gaynor et al. [13] conducted a stochastic simulation and showed that programs using the two-part strategy generated up to 2.5- and 1.5-fold more genetic gain than conventional programs and the best performing standard genomic selection strategy, respectively. Additionally, Yao et al. [14] combined GP with Monte Carlo simulation to select superior parents in wheat breeding programs before field trials. The authors used the criterion of usefulness function on a selection index, which incorporates yield and two quality traits, to evaluate a cross, and concluded that the use of the usefulness function for parental selection resulted in higher genetic gain than the use of mid-parent GEBV, implying that the strategy for parental selection cannot only consider GEBVs of the candidate accessions.

By selecting parental lines with the highest GEBVs, breeders hope to maximally pass the favorable traits of parental lines on to their progeny. However, the truncation selection approach risks the elimination of several favorable QTLs from the breeding population because of a lack of genomic diversity [15]. Therefore, in this study, we took both GEBV and genomic diversity into account for identifying superior parents in a biparental crossing program. We constructed a GBLUP model for a specific target trait to predict the GEBVs of the candidate accessions. We first proposed a new index to quantify the genomic diversity of a set of candidate accessions. Subsequently, we simulated the genotypic data for progeny populations derived from a cross over successive generations, and predicted the GEBVs of the simulated progeny populations through the trained GBLUP model. Then, we made generation advancement decisions according to the resulting GEBVs. Finally, we assessed a set of parental lines based on F_10_ RILs. We compared the performance of several selection strategies via analysis of two real rice genome datasets.

## Materials and methods

### Rice genome datasets

#### Dataset I

**Fig 1:**
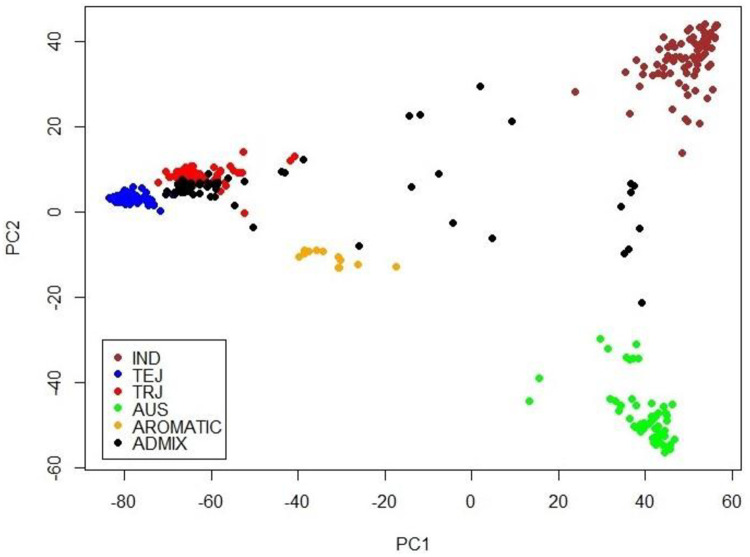
Scatter plot of 413 accessions with 11,047 SNPs according to the first two principal components (PCs) for the 44k rice genome dataset. IND: *indica* rice; TEJ: temperate *japonica* rice; TRJ: tropical *japonica* rice; AUS: Aus rice; AROMATIC: aromatic rice; ADMIX: admixed group.

The rice genome dataset originally collected for genome-wide association study (GWAS) in Zhao et al. [16] was used to illustrate the proposed procedure. This dataset contains 44,100 SNP variants and 36 traits of 413 *O*. *sativa* accessions, which comprises five subpopulations and one admixed group. SNPs with missing rate > 0.05 and minor allele frequency < 0.05 were removed from the dataset. To reduce redundant collinearity in the genomic relationship matrix, one SNP was randomly selected from each bin of 20,000 bp over each chromosome. A scatter plot based on the first two principal components (PCs) using the retained 11,047 SNPs is displayed in Fig 1, which is almost the same as that in the corresponding plot generated using all 44,100 SNPs [16]. The SNP genotype at each locus was coded as -1, 0, or 1, where 1 indicates homozygous genotype of the major allele; -1 indicates homozygous genotype of the minor allele; and 0 indicates heterogenous genotype. After SNP coding, any missing locus was imputed as 1. Six traits were analyzed: brown rice seed width (BRSW), florets per panicle (FPP), flowering time at Arkansas (FTAA), flowering time at Faridpur (FTAF), plant height (PH), and panicle number per plant (PNPP).

#### Dataset II

The rice genome dataset, which was collected for genomic selection study [8], was further analyzed as dataset II. This dataset contains 73,147 SNP variants and 363 elite breeding lines belonging to *indica* or *indica*–admixed group. Phenotypic observations include four years (2009–2012; two seasons per year [dry and wet]) of data on grain yield (YLD), flowering time (FT), and plant height (PH), although PH data in the wet season of 2009 were not available. Phenotypic values of 35 out of 363 individuals were missing; therefore, adjusted means of only 328 individuals were used in this study. Additionally, only 10,772 out of 73,147 SNPs were used in this study. One SNP marker was selected per 0.1-cM interval on each chromosome because the chosen subset of the full marker set has been shown to be efficient enough for genomic selection in this collection of rice germplasm [8].

### Monte Carlo simulation of the genotypic data of progeny populations

To simulate the genotypic data of progeny populations, the Gramene Annotated Nipponbare Sequence [17] was used to estimate recombination rates between two adjacent SNPs. The Gramene Annotated Nipponbare Sequence database contains both physical and linkage distances between SNPs, which can be downloaded from http://archive.gramene.org. The genetic positions of SNPs were estimated via linear interpolation between the two markers flanking each SNP. Once the genetic positions were obtained, the recombination rates between adjacent SNPs were estimated using Haldane’s mapping function [18]:

$$r_{AB}=\frac{1}{2}(1-e^{-2X_{AB}})$$

where *r*_*AB*_ is the recombination rate between markers A and B; *X*_*AB*_ is the linkage distance between markers A and B; and *e* is Euler’s number, a mathematical constant approximately equal to 2.71828. Based on a series of Bernoulli distributions and the estimated recombination rates, the crossover of each chromosome was simulated to yield the sequence of a gamete. Then, two gametes were paired to produce the genotypic data for the progeny.

### GBLUP model

The following GBLUP model was considered for GP:

$$y=\mu 1_{n}+g+e$$ (1)

where **y** denotes the vector of phenotypic values of a training population with *n* individuals; *μ* is a constant term; **1**_*n*_ is the vector of order *n* with all elements equal to 1; ***g*** represents the vector of genotypic values; and ***e*** is the vector of random errors.

It is assumed that ***g*** follows a multivariate normal distribution $MVN(0,\sigma_{g}^{2}K)$, where **0** is a zero vector; $\sigma_{g}^{2}$ is the genetic variance of additive effects; and ***K*** is a genomic relationship matrix among the individuals. Furthermore, ***e*** follows $MVN(0,\sigma_{e}^{2}I_{n})$, where $\sigma_{e}^{2}$ is the random error variance, and ***I***_*n*_ denotes the identity matrix of order *n*. Here, ***g*** and ***e*** are assumed to be mutually independent. In this study, the genomic relationship matrix ***K*** = ***MM***^*T*^/*p* was considered, where ***M*** is the marker score matrix, and *p* is the number of SNP markers.

The model parameters of the GBLUP model can be estimated through the Henderson’s equation [19], as follows:

$$\left[\begin{array}{ll} n & 1_{n}^{T}\\ 1_{n} & I_{n}+\lambda K^{-1} \end{array}][\begin{array}{l} \hat{\mu }\\ \hat{g} \end{array}]=[\begin{array}{l} 1_{n}^{T}y\\ y \end{array}\right]$$ (2)

where the regularization parameter *λ* is given by $\lambda =\frac{\sigma_{e}^{2}}{\sigma_{g}^{2}}$.

The mmer () function in the R package sommer [20] was used to obtain the restricted maximum likelihood estimates (REMLs) for the two variance components of $\sigma_{g}^{2}$ and $\sigma_{e}^{2}$, and the resulting estimates were entered into Eq (2) to obtain $\hat{\mu }$ and $\hat{g}$.

If ${\hat{g}}_{bp}$ is considered as the vector of estimated genotypic values for a breeding population, and ***K***_*bp*_ is considered as the genomic relationship matrix between the breeding and training populations, the following equation is obtained:

$${\hat{g}}_{bp}=K_{bp}K^{-1}\hat{g}$$

The GEBV for the breeding population is ${\hat{g}}_{bp}$ plus the estimate of the constant term $\hat{\mu }$.

### Index for quantifying genomic diversity

Let ***g***_0_ be the vector of genotypic values, and ***K***_0_ be the genomic relationship matrix for a particular set of accessions with size *n*_0_. According to the GBLUP model in Eq (1), the covariance matrix for ***g***_0_ is given by:

$$Var(g_{0})=\sigma_{g}^{2}K_{0}$$

The determinant of the covariance matrix represents the overall variability of the genotypic values, which is calculated as:

$$\left|Var(g_{0})|={\left(\sigma_{g}^{2}\right)}^{n_{0}}|K_{0}\right|$$ (3)

Clearly, the determinant of Eq (3) is proportional to the *D*-score defined below:

$$D-\text{score}=|K_{0}|$$ (4)

For a fixed number of *n*_0_, a subset of accessions chosen from a breeding population with the maximal *D*-score will have greater genomic diversity than the competing choices with size *n*_0_. The concept of the *D-*score is adopted from optimum experimental designs [21].

A simple example is given below to illustrate the *D-*score. Suppose that there are three accessions (*n* = 3) in the candidate set with the genomic relationship matrix:

$$K=[\begin{array}{ccc} 1 & 0.9 & 0.5\\ 0.9 & 1 & 0.1\\ 0.5 & 0.1 & 1 \end{array}]$$

For *n*_0_ = 2, the *D-*score for *g*_1_ and *g*_2_ is calculated as $|K_{0}|=|\begin{array}{cc} 1 & 0.9\\ 0.9 & 1 \end{array}|=0.19$. Similarly, the *D*-scores for *g*_1_ and *g*_3_ and for *g*_2_ and *g*_3_ are given as 0.75 and 0.99, respectively. Clearly, the two accessions with *g*_2_ and *g*_3_ genotypic values have greater genomic variation (smaller genomic correlation) than the other competing choices. Closely related individuals could be excluded from the maximal *D-*score set. The genetic algorithm presented in Ou and Liao [22] was used to search a subset of accessions from a candidate population, such that it can attain the maximal *D-*score of Eq (4).

### Procedure for selecting parental lines

To evaluate a variety of strategies for determining parental lines, the following steps were carried out:

1. For a specific target trait, all phenotypic values available from each rice genome dataset were used to build the corresponding GBLUP model of Eq (1).
2. The GEBVs of all accessions in the dataset were predicted through the trained GBLUP model in step 1. Seven strategies were used to select a subset of 10 parental lines according to their GEBVs: (i) the GEBV only (GEBV-O) approach, which chose the top 10 accessions (either maximal or minimal); three genomic diversity only (GD-O) approaches: (ii) GD-O-30, (iii) GD-O-50, and (iv) GD-O-100, which applied the genetic exchange algorithm to search for an optimal subset of 10 accessions from each of the three candidate sets composed of the top 30, 50, and 100 accessions, respectively, such that the chosen subset had the maximal *D*-score; and three approaches that considered both GEBV and genomic diversity: (v) GEBV-GD-30, (vi) GEBV-GD-50, and (vii) GEBV-GD-100, which retained the top two accessions, and then applied the genetic exchange algorithm to search for another eight accessions from the remainder of each candidate set for GD-O-30, GD-O-50, and GD-O-100, respectively, so that the resulting 10 accessions had the maximal *D*-score.
3. For each subset of 10 accessions determined by the seven strategies, any two parental lines were crossed to produce 45 F_1_ hybrids. Here, we started to simulate the genotypic data for successive generations of progeny populations through the Monte Carlo simulation. Each of the 45 F_1_ hybrids produced 60 individuals of the F_2_ population by self-pollination, resulting in 2,700 F_2_ individuals. After obtaining the GEBVs for the 2,700 F_2_ individuals via the trained GBLUP model, the top 45 F_2_ individuals were retained. Again, these 45 F_2_ individuals were used to produce 2,700 F_3_ individuals (60 F_3_ individuals per F_2_ individual), and the top 45 F_3_ individuals were retained. The same procedure was repeated to produce 2,700 F_10_ individuals, which were assumed to be a fixed population.
4. For the resulting 2,700 F_10_ individuals generated according to each strategy, we found the best F_10_ RILs with the highest GEBVs.

**Fig 2:**
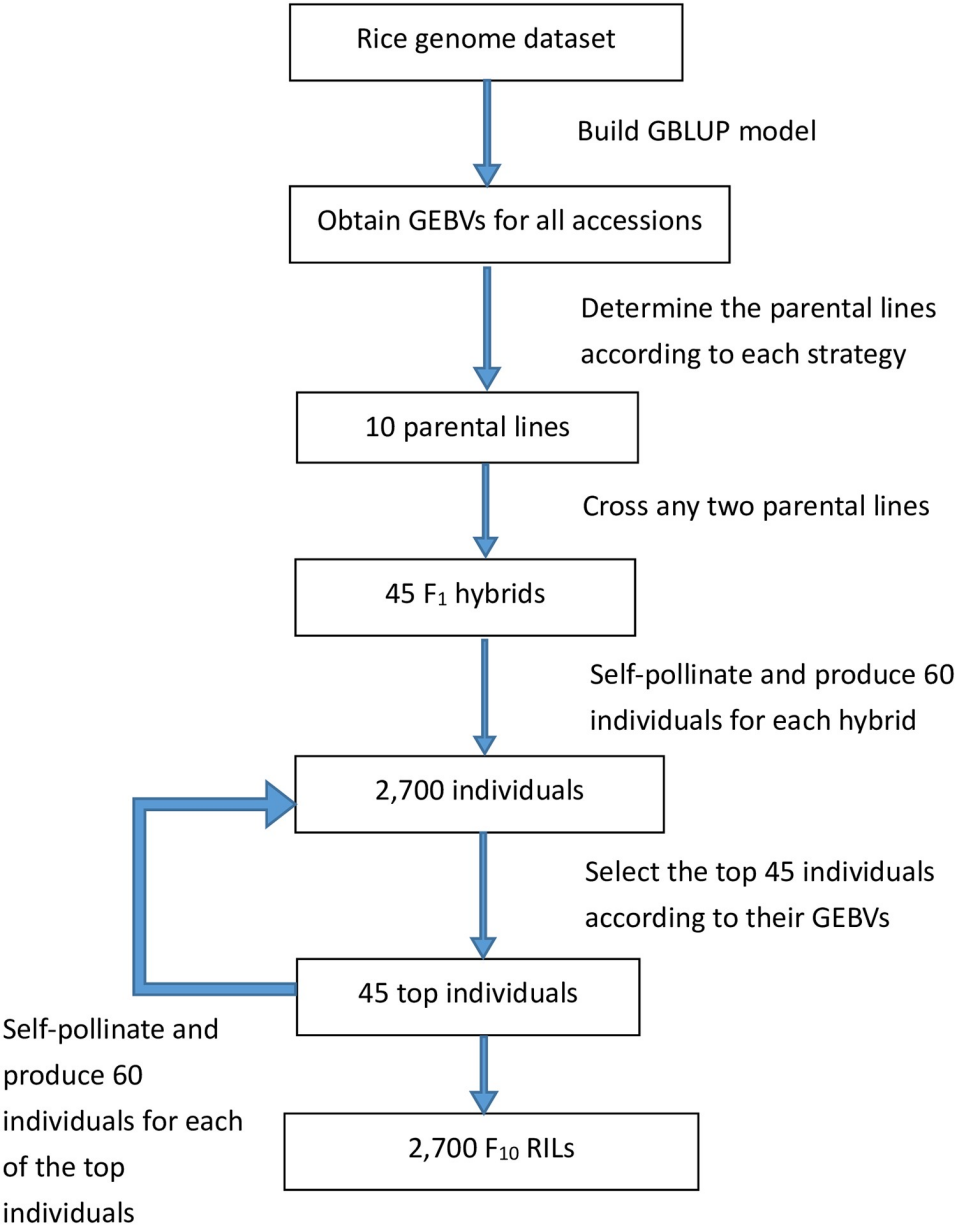
Flowchart showing the Monte Carlo simulation. GEBV: genomic estimated breeding value; GBLUP: genomic best linear unbiased predictor; RIL: recombinant inbred line.

A flowchart of this procedure is displayed in Fig 2. This procedure was repeated 30 times to obtain the best F_10_ RILs from each repetition for each strategy. The average of the GEBVs for the best F_10_ RILs was then calculated and used as the measure of efficiency for the strategy. Then, pairwise comparisons were performed among the GEBV averages, based on the least significant difference (LSD) test. Note that for BRSW, FPP, and PNPP in Dataset I and for YLD in Dataset II, larger GEBVs are preferable (i.e., for these traits, the larger the GEBV, the better). The remaining five traits (FTAA, FTAF, and PH in Dataset I, and FT and PH in Dataset II) follow the rule: that the smaller the GEBV, the better.

### Calculation of genetic gain

To understand the genetic improvement in a target trait using different strategies, the genetic gain was estimated as:

$${Genetic\;gain=\overset{-}{GEBV}}_{F_{10}}-{\overset{-}{GEBV}}_{P}$$ (5)

where ${\overset{-}{GEBV}}_{F_{10}}$ denotes the average GEBV of the resulting 2,700 F_10_ RILs; and ${\overset{-}{GEBV}}_{P}$ denotes the average GEBV of 10 parental lines selected using each strategy [23]. The larger the absolute value of the genetic gain, the greater the improvement in the target trait. The average of genetic gains from 30 repetitions was reported for each strategy, and multiple comparisons among the genetic gain averages were performed using the LSD test.

## Results

### Comparison of strategies based on the best F_10_ RILs

The GEBV averages of the best F_10_ RILs and results of the LSD test are displayed in Tables 1 and 2 for Datasets I and II, respectively. The strategies that considered both GEBV and genomic diversity (GEBV-GD-30, -50, -100) generally showed satisfactory efficiency because they achieved the best or second-best performance for all traits. Therefore, these types of strategies could be used as a reliable means for selecting parental lines. On the other hand, strategies accounting for only genomic diversity (GD-O-30, -50, -100) did not show satisfactory efficiency for all traits, except GD-O-100, which was satisfactory for YLD in Dataset II. The GEBV-O strategy showed the best or second-best performance for FPP and PH in Dataset I and for PH and FT in Dataset II, but it also showed the worst or second-worst performance for the remaining four traits in Dataset I and for YLD in Dataset II. These data indicate that GEBV-O is a high-risk strategy. In general, the results of the LSD test showed significant differences in GEBV averages between the best/second-best and worst/second-worst performances for all traits in both datasets.

**Table 1:** Ranking and GEBV averages (in parentheses) of the best F_10_ RILs selected by 30 repetitions of the seven proposed strategies applied to six traits in Dataset I.

Strategy^1^	Traits^2^					
	BRSW	FPP	FTAA	FTAF	PH	PNPP
GEBV-O	6 (3.418)e	**2 (5.961)**a	6 (56.52)e	6 (61.85)d	**1 (42.18)**a	6 (4.125)c
GD-O-30	7 (3.408)e	5 (5.951)a	3 (51.56)b	3 (59.35)a	5 (49.33)b	3 (4.188)b
GD-O-50	3 (3.576)c	6 (5.916)b	5 (53.34)d	5 (60.12)c	6 (49.80)b	5 (4.138)c
GD-O-100	4 (3.496)d	7 (5.882)c	7 (56.83)e	7 (61.96)d	7 (51.78)c	7 (4.086)d
GEBV-GD-30	5 (3.419)e	3 (5.954)a	**1 (47.13)**a	**1 (59.21)**a	**2 (42.69)**a	**1 (4.225)**a
GEBV-GD-50	**1 (3.656)**a	**1 (5.964)**a	**2 (47.45)**a	**2 (59.30)**a	3 (43.23)a	**2 (4.214)**a
GEBV-GD-100	**2 (3.634)**b	4 (5.953)a	4 (51.38)c	4 (59.63)b	4 (43.49)a	4 (4.171)b

^1^ GEBV-O: subset of the top 10 accessions with minimal or maximal GEBV; GD-O-30, -50, -100: subsets of 10 accessions with the maximal D-scores chosen from candidate sets comprising the top 30, 50, and 100 accessions, respectively; GEBV-GD-30, -50, -100: subsets of the top two accessions plus eight accessions chosen from the remainder of the candidate sets composed of the top 30, 50, and 100 accessions, respectively, with the maximal D-scores.

^2^ BRSW: brown rice seed width; FPP: florets per panicle; FTAA: flowering time at Arkansas; FTAF: flowering time at Faridpur; PH: plant height; PNPP: panicle number per plant. Different lowercase letters indicate significant differences among the strategies for a given trait (*P* < 0.01; LSD test). The best and second-best strategies are indicated in bold, while the worst and second-worst strategies are underlined.

**Table 2:** Ranking and GEBV averages (in parentheses) of the best F_10_ RILs selected by 30 repetitions of the seven proposed strategies applied to three traits in Dataset II.

Strategy^1^	Traits^2^		
	YLD	PH	FT
GEBV-O	7 (6472)b	**1 (85.817)**a	**2 (77.818)**a
GD-O-30	4 (6491)b	5 (87.517)b	7 (78.410)c
GD-O-50	5 (6489)b	6 (89.920)c	5 (78.164)b
GD-O-100	**1 (6546)**a	7 (91.799)e	6 (78.359)bc
GEBV-GD-30	3 (6506)ab	3 (85.976)a	4 (77.883)a
GEBV-GD-50	6 (6485)b	**2 (85.917)**a	**1 (77.725)**a
GEBV-GD-100	**2 (6539)**a	4 (86.062)a	3 (77.873)a

^1^ GEBV-O: subset of the top 10 accessions with the minimal or maximal GEBV; GD-O-30, -50, -100: subsets of 10 accessions with maximal D-scores chosen from the candidate sets comprising the top 30, 50, and 100 accessions, respectively; GEBV-GD-30, -50, -100: subsets of the top two accessions plus eight accessions chosen from the remainder of the candidate sets composed of the top 30, 50, and 100 accessions, respectively, with the maximal D-scores.

^2^ YLD: yield; PH: plant height; FT: flowering time. Different lowercase letters indicate significant differences among the strategies for a given trait (*P* < 0.01; LSD test). The best and second-best strategies are indicated in bold, while the worst and second-worst strategies are underlined.

**Fig 3:**
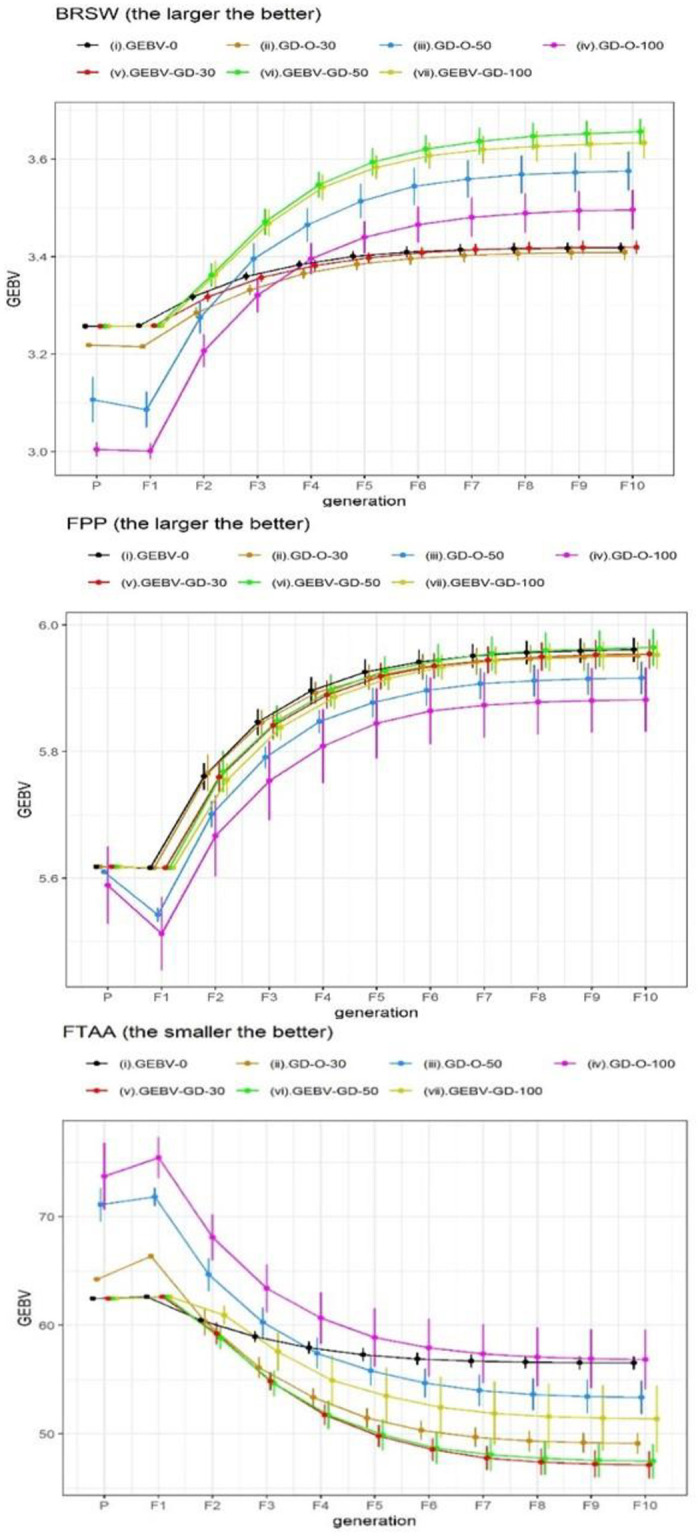
GEBV averages of the best individuals selected from 30 repetitions at consecutive generations for the six chosen traits in Dataset I. GEBV-O: subset of the top 10 accessions with minimal or maximal GEBVs; GD-O-30, -50, -100: subsets of 10 accessions with maximal D-scores chosen from candidate sets composed of the top 30, 50, and 100 accessions, respectively; GEBV-GD-30, -50, -100: subsets of the top two accessions plus eight accessions chosen from the remainder of the candidate sets composed of the top 30, 50, and 100 accessions, respectively, with maximal D-scores; BRSW: brown rice seed width; FPP: florets per panicle; FTAA: flowering time at Arkansas.

**Fig 4:**
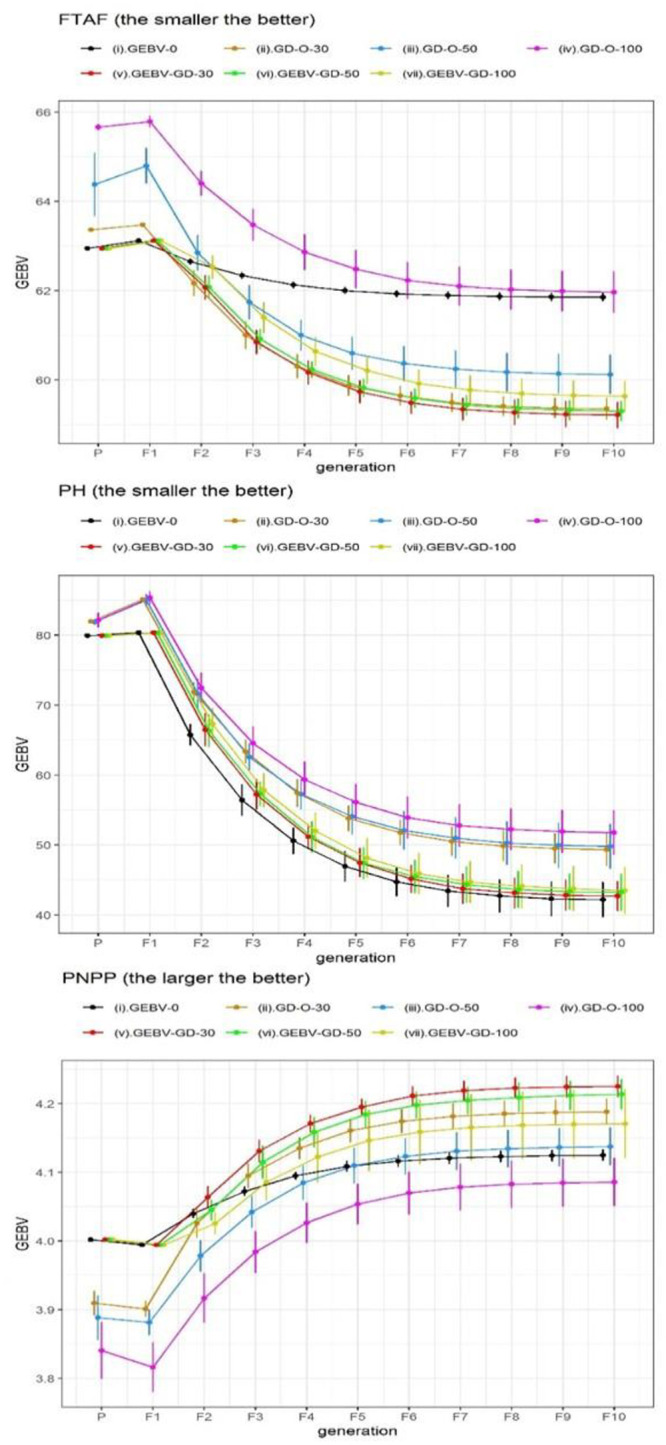
GEBV averages of the best individuals selected from 30 repetitions at consecutive generations for the six chosen traits in Dataset I. GEBV-O: subset of the top 10 accessions with minimal or maximal GEBVs; GD-O-30, -50, -100: subsets of 10 accessions with maximal D-scores chosen from candidate sets composed of the top 30, 50, and 100 accessions, respectively; GEBV-GD-30, -50, -100: subsets of the top two accessions plus eight accessions chosen from the remainder of the candidate sets composed of the top 30, 50, and 100 accessions, respectively, with maximal D-scores; FTAF: flowering time at Faridpur; PH: plant height; PNPP: panicle number per plant.

**Fig 5:**
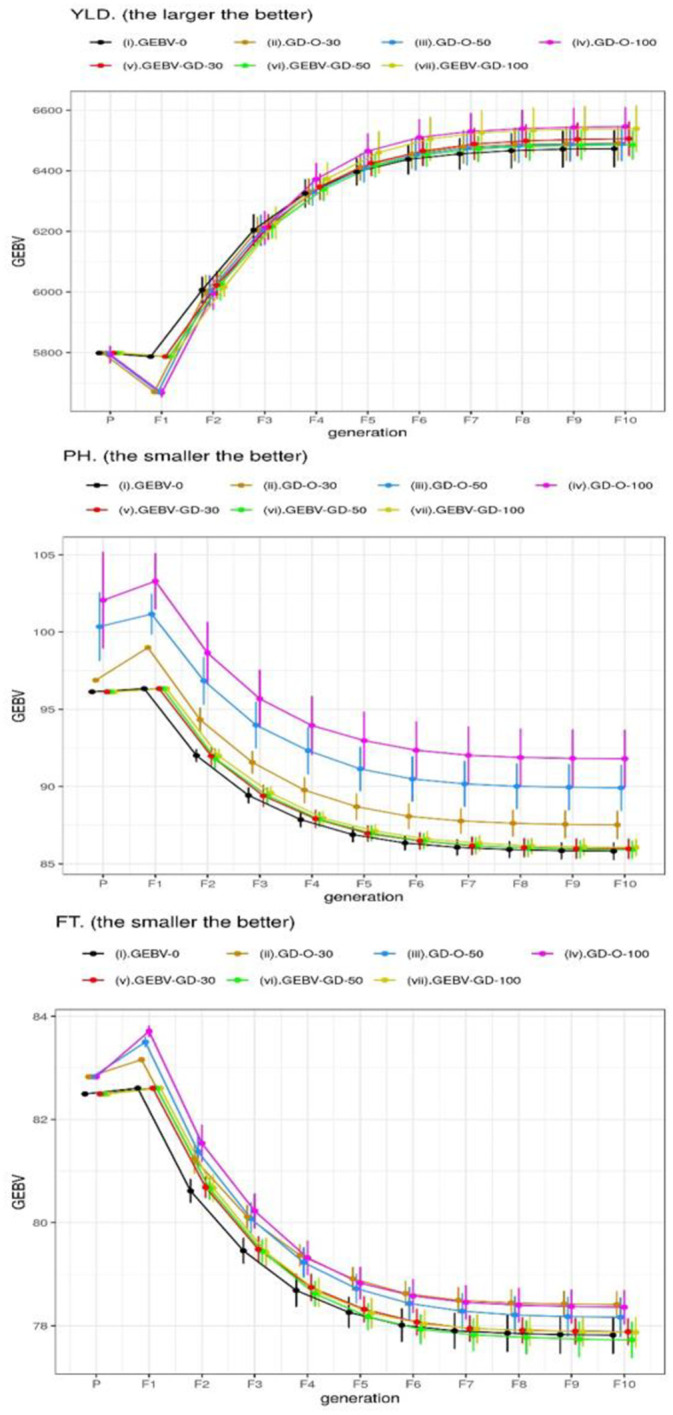
GEBV averages of the best individuals selected from 30 repetitions at consecutive generations for the three target traits in Dataset II. GEBV-O: subset of the top 10 accessions with minimal or maximal GEBVs; GD-O-30, -50, -100: subsets of 10 accessions with maximal D-scores chosen from candidate sets composed of the top 30, 50, and 100 accessions, respectively; GEBV-GD-30, -50, -100: subsets of the top two accessions plus eight accessions chosen from the remainder of the candidate sets composed of the top 30, 50, and 100 accessions, respectively, with the maximal D-scores; YLD, yield; PH, plant height; FT, flowering time.

We also displayed the average GEBV ± standard deviation (SD) of the best RILs selected by 30 repetitions over consecutive generations in Figs 3, 4 and 5. Four strategies including GEBV-O, GEBV-GD-30, -50, and -100 selected the same best individual from the 30 repetitions at the parental generation and also at the F_1_ generation; therefore, no SD is shown with the corresponding GEBV averages. The GEBV averages of the best parental lines selected by the strategies can be ranked as GEBV-O = GEBV-GD-30 = GEBV-GD-50 = GEBV-GD-100 ≥ GD-O-30 ≥ GD-O-50 ≥ GD-O-100 in decreasing desirability. The desirability at the parental generation decreased as the degree of diversity increased for the three strategies, considering the genomic diversity only. Additionally, the desirability declined from the parental generation to F_1_ generation for every strategy because of the heterozygous alleles in F_1_ hybrids.

To explore the extent to which the top two accessions contributed to the subset of 10 parental lines determined by four strategies (GEBV-O, GEBV-GD-30, -50, and -100), we compared each subset with a reduced group consisting of F_1_ hybrids, whose parental lines contained at least one of the top two accessions for each subset. Each reduced group consisted of 17 F_1_ hybrids. Similarly, we followed the analysis procedure to obtain the GEBV averages of the best F_10_ RILs from 30 repetitions based on the reduced group. The results are displayed in Table 3, with the corresponding GEBV averages based on the group of the original 45 F_1_ hybrids. The results showed no practical significance between these two groups for all the traits using the four strategies (Table 3). Therefore, the reduced group can be an alternative to the full group.

**Table 3:** GEBV averages of the best F_10_ RILs selected by 30 repetitions, based on the original group comprising 45 F_1_ hybrids and the reduced group comprising 17 F_1_ hybrids, using four strategies.

Trait^1^	Strategy^2^							
	GEBV-O		GEBV-GD-30		GEBV-GD-50		GEBV-GD-100	
Dataset I	45 F_1_	17 F_1_	45 F_1_	17 F_1_	45 F_1_	17 F_1_	45 F_1_	17 F_1_
BRSW	3.418	3.423	3.419	3.418	3.656	3.652	3.634	3.650
FPP	5.961	5.965	5.954	5.957	5.964	5.958	5.953	5.943
FTAA	56.521	57.513	47.136	46.961	47.457	47.421	51.382	51.734
FTAF	61.856	61.850	59.216	59.123	59.304	59.232	59.634	59.713
PH	42.185	43.409	42.699	43.271	43.232	43.791	43.498	43.854
PNPP	4.125	4.129	4.225	4.226	4.214	4.204	4.171	4.161
Dataset II	45 F_1_	17 F_1_	45 F_1_	17 F_1_	45 F_1_	17 F_1_	45 F_1_	17 F_1_
YLD	6472	6476	6506	6499	6485	6484	6539	6534
PH	85.817	85.991	85.976	85.844	85.917	86.092	86.062	86.060
FT	78.818	77.834	77.883	77.750	77.725	77.778	77.873	77.690

^1^ BRSW: brown rice seed width; FPP: florets per panicle; FTAA: flowering time at Arkansas; FTAF: flowering time at Faridpur; PH: plant height; PNPP: panicle number per plant; YLD: yield; PH: plant height; FT: flowering time.

^2^ GEBV-O: subset of the top 10 accessions with minimal or maximal GEBVs; GEBV-GD-30, -50, -100: subsets of the top two accessions plus eight accessions chosen from the remainder of the candidate sets composed of the top 30, 50, and 100 accessions, respectively, with the maximal D-scores.

### Genetic gains with different strategies

The average genetic gains and results of the LSD test are displayed in Tables 4 and 5 for Datasets I and II, respectively. It is also reasonable to evaluate the performance of the strategies according to the endpoint of ${\overset{-}{GEBV}}_{F_{10}}$. The comparison results based on ${\overset{-}{GEBV}}_{F_{10}}$ were consistent with the above comparison results based on the best F_10_ RILs. Strategies that considered genomic diversity (GD-O-30, -50, -100; GEBV-GD-30, -50, -100) showed greater genetic gain than the GEBV-O for all traits, except PH in Dataset I (Table 4). The genetic gain generally increased with the increase in genomic diversity, as expected (GD-O-100 outperformed both GD-O-50 and GD-O-30 for all traits, except BRSW and FTAF in Dataset I; GEBV-GD-100 outperformed both GEBV-GD-50 and GEBV-GD-30 for all traits). The results of the LSD test showed that the GEBV-GD-100 strategy significantly differs from the remaining strategies in genetic gain for all traits in Dataset I, but it showed no significant difference from GEBV-GD-50 for FTAA and from GEBV-GD-50 and -30 for PH. On the other hand, the GD-O-100 strategy significantly differed from the remaining strategies for all traits in Dataset II, except from the GEBV-GD-100 for PH. In addition, GEBV-O showed the best ${\overset{-}{GEBV}}_{P}$, while GEBV-GD-30, -50, and -100 showed higher ${\overset{-}{GEBV}}_{P}$ than their counterparts (GD-O-30, -50, and -100, respectively) for all traits in both datasets. Thus, a strategy has a relatively good starting point, as it considers more candidate accessions with the top GEBVs.

**Table 4:** Average genetic gains from 30 repetitions for Dataset I.

Strategy^1^	Brown rice seed width (BRSW)			Florets per panicle (FPP)		
	${\overset{-}{GEBV}}_{P}$^2^	${\overset{-}{GEBV}}_{F_{10}}$^3^	Genetic gain^4^	${\overset{-}{GEBV}}_{P}$	${\overset{-}{GEBV}}_{F_{10}}$	Genetic gain
GEBV-O	3.17	3.42	0.25f	5.51	5.96	0.45f
GD-O-30	3.10	3.41	0.31e	5.48	5.95	0.47e
GD-O-50	3.00	3.57	0.57c	5.41	5.91	0.50d
GD-O-100	2.94	3.49	0.55d	5.31	5.88	0.57b
GEBV-GD-30	3.12	3.42	0.30e	5.48	5.95	0.47e
GEBV-GD-50	3.04	3.65	0.61b	5.43	5.96	0.53c
GEBV-GD-100	3.00	3.63	0.63a	5.34	5.95	0.61a
	Flowering time at Arkansas (FTAA)			Flowering time at Faridpur (FTAF)		
	${\overset{-}{GEBV}}_{P}$	${\overset{-}{GEBV}}_{F_{10}}$	Genetic gain	${\overset{-}{GEBV}}_{P}$	${\overset{-}{GEBV}}_{F_{10}}$	Genetic gain
GEBV-O	64.30	56.57	-7.73d	63.45	61.87	-1.58f
GD-O-30	72.25	49.26	-22.99bc	64.93	59.40	-5.53cd
GD-O-50	75.41	53.54	-21.87c	65.82	60.16	-5.66c
GD-O-100	80.01	57.00	-23.01bc	67.34	62.01	-5.33e
GEBV-GD-30	71.09	47.31	-23.78b	64.68	59.25	-5.43de
GEBV-GD-50	72.86	47.64	-25.22a	65.40	59.35	-6.05b
GEBV-GD-100	77.16	51.53	-25.63a	66.46	59.68	-6.78a
	Plant height (PH)			Panicle number per plant (PNPP)		
	${\overset{-}{GEBV}}_{P}$	${\overset{-}{GEBV}}_{F_{10}}$	Genetic gain	${\overset{-}{GEBV}}_{P}$	${\overset{-}{GEBV}}_{F_{10}}$	Genetic gain
GEBV-O	83.77	42.52	-41.25b	3.93	4.12	0.19e
GD-O-30	89.50	49.69	-39.81b	3.86	4.19	0.33d
GD-O-50	90.11	50.13	-39.98b	3.80	4.14	0.34d
GD-O-100	92.10	52.10	-40.00b	3.64	4.08	0.44b
GEBV-GD-30	87.26	42.99	-44.27a	3.90	4.22	0.32d
GEBV-GD-50	87.95	43.50	-44.45a	3.84	4.21	0.37c
GEBV-GD-100	89.27	43.95	-45.32a	3.70	4.17	0.47a

^1^ GEBV-O: subset of the top 10 accessions with minimal or maximal GEBVs; GD-O-30, -50, -100: subsets of 10 accessions with maximal D-scores chosen from the candidate sets composed of the top 30, 50, and 100 accessions, respectively; GEBV-GD-30, -50, -100: subsets of the top two accessions plus eight accessions chosen from the remainder of the candidate sets composed of the top 30, 50, and 100 accessions, respectively, with the maximal D-scores.

^2^ ${\overset{-}{GEBV}}_{P}$: average GEBV of the 10 selected parental lines.

^3^ ${\overset{-}{GEBV}}_{F_{10}}$: average GEBV of 2,700 F_10_ RILs.

^4^ Lowercase letters indicate significant differences among strategies for a given trait (*P* < 0.01; LSD test).

**Table 5:** Average genetic gains derived from 30 repetitions for Dataset II.

Strategy^1^	Yield (YLD)		
	${\overset{-}{GEBV}}_{P}$^2^	${\overset{-}{GEBV}}_{F_{10}}$^3^	Genetic gain^4^
GEBV-O	5571.61	6468.60	896.99e
GD-O-30	5452.39	6488.02	1035.63c
GD-O-50	5436.58	6484.58	1048.00bc
GD-O-100	5289.74	6540.72	1250.98a
GEBV-GD-30	5538.44	6501.23	962.79d
GEBV-GD-50	5522.45	6482.13	959.68d
GEBV-GD-100	5454.37	6535.79	1081.42b
	Plant height (PH)		
	${\overset{-}{GEBV}}_{P}$	${\overset{-}{GEBV}}_{F_{10}}$	Genetic gain
GEBV-O	97.75	85.89	-11.86d
GD-O-30	102.20	87.59	-14.61a
GD-O-50	103.66	89.99	-13.67b
GD-O-100	106.83	91.85	-14.98a
GEBV-GD-30	99.00	86.01	-12.99c
GEBV-GD-50	99.39	85.99	-13.40bc
GEBV-GD-100	101.15	86.13	-15.02a
	Flowering time (FT)		
	${\overset{-}{GEBV}}_{P}$	${\overset{-}{GEBV}}_{F_{10}}$	Genetic gain
GEBV-O	83.14	77.84	-5.30e
GD-O-30	83.98	78.43	-5.55d
GD-O-50	84.57	78.19	-6.38b
GD-O-100	85.62	78.39	-7.23a
GEBV-GD-30	83.44	77.90	-5.54d
GEBV-GD-50	83.69	77.76	-5.93c
GEBV-GD-100	84.16	77.89	-6.27b

^1^ GEBV-O: subset of the top 10 accessions with minimal or maximal GEBVs; GD-O-30, -50, -100: subsets of 10 accessions with maximal D-scores chosen from the candidate sets composed of the top 30, 50, and 100 accessions, respectively; GEBV-GD-30, -50, -100: subsets of the top two accessions plus eight accessions chosen from the remainder of the candidate sets composed of the top 30, 50, and 100 accessions, respectively, with the maximal D-scores.

^2^ ${\overset{-}{GEBV}}_{P}$: average GEBV of the 10 selected parental lines.

^3^ ${\overset{-}{GEBV}}_{F_{10}}$: average GEBV of 2,700 F_10_ RILs.

^4^ Lowercase letters indicate significant differences among the strategies for a given trait (*P* < 0.01; LSD test).

## Discussion

Dataset II was specifically collected for genomic selection. All the available accessions in this dataset are *indica* or *indica–*admixed. The results of performance based on the best F_10_ RILs (Table 2) revealed that all seven strategies showed similar performance for the three target traits. The resulting GEBV averages of the best F_10_ RILs ranged from 6472 to 6546 kg/ha for YLD, from 85.889 to 91.852 cm for PH, and from 77.725 to 78.410 days for FT. This could be because the candidate accessions in Dataset II are elite breeding lines, with limited genetic diversity and similar phenotypic values for the target traits. However, the results of the LSD test revealed that the two strategies (GD-O-100 and GEBV-GD-100) with greater genomic diversity for YLD led to significantly larger YLD than the other five strategies. Four strategies including GEBV-O, GEBV-GD-30, -50, and -100 performed equally well for PH but performed significantly better than GD-O-30, -50, and -100.

It is well known that Dataset I contains more genomic diversity than Dataset II since it consists of five subpopulations and one admixed group. The higher genomic diversity of Dataset I could result in a bigger difference between GEBV-GD-30/50/100 strategies and the GEBV-O strategy for some traits. For example, the difference in the GEBV averages among the best F_10_ RILs between GEBV-GD-50 and GEBV-O was approximately -9.06 days for FTAA and -2.55 days for FTAF in Dataset I (Table 1), but the corresponding difference was only -0.09 days for FT in Dataset II (Table 2). However, the flowering time is very sensitive to environmental conditions, implying that genomic diversity cannot solely amount to the differences in results between these two datasets. More interestingly, the higher genomic diversity of Dataset I could lead to a larger genetic gain for a specific trait. The average genetic gain using the seven strategies for PH in Dataset I was -42.15 cm (Table 4); however, the corresponding mean in Dataset II was only -13.79 cm (Table 5).

The average GEBV of the best F_10_ RILs for YLD was the highest using the GD-O-100 strategy on Dataset II (Table 2). However, the corresponding GEBV averages for two yield components, FPP and PNPP, were the lowest in Dataset I (Table 1). This is possible because the analysis results were based on two different collections of rice lines. There is little diversity among the RILs in Dataset II; therefore, the difference in the average GEBV for YLD among the strategies seems to be negligible. Note that the LSD test revealed only two significance groups in YLD. Nonetheless, the results of FPP and PNPP analysis using the GD-O-100 strategy in Dataset I appear to be reasonable.

Apparently, the number of accessions fixed in the proposed strategies seemed to be arbitrary, similar to the selection of 10 parental lines, retaining the top 2 accessions, and searching 10 or another 8 accessions from the three candidate sets composed of the top 30, 50, and 100 accessions, respectively. A user certainly can adjust these numbers in the strategies according to their own study. Additionally, historical phenotypic data were required to build the GP model. If the historical phenotypic data are not available, then a pilot experiment is needed to phenotype a set of accessions, which can be determined using an optimization algorithm [22]. Two R functions used to perform the proposed procedure for selecting parental lines are provided in S1 File.

We addressed the issue that incorporating genomic diversity into the conventional truncation selection could improve the likelihood of identifying superior parental lines. More importantly, we showed that combining GP with Monte Carlo simulation could help breeders to discover superior parental lines before conducting field experiments. It is well known that phenotype is affected by the genotype (G), environment (E), and G × E interaction. In reality, environment can have a significant impact on the performance of progeny populations during the growth period of each generation until reaching the F_10_ generation. Thus, parental lines selected from our simulation study may not perform as expected. Therefore, conducting field experiments to validate our study would be worthwhile. As mentioned earlier, the works of Gaynor et al. [13] and Yao et al. [14] support that the strategy for selecting better parental lines through GP with Monte Carlo simulation should prove useful in plant breeding. The study of Vanavermaete et al. [15] supports our theory that considering both GEBV and genomic diversity in parental selection is a promising strategy.

In this study, we focused on single-trait selection; therefore, the proposed approach could select different parental lines for different target traits. In practice, it is desirable to extend the approach to multiple-trait selection. A straightforward extension is to apply a selection index incorporating multiple traits, and then treat the selection index as a new target trait for the current single-trait approach. Another possible modification is to directly implement multiple-trait GP models, and then use an appropriate selection index for evaluating candidate accessions. Jia and Jannink [24], Hayashi and Iwata [25], and Guo et al. [26] have shown that multiple-trait GP models provide better prediction accuracy than single-trait GP models for a low-heritability trait, which shows strong correlation with a high-heritability trait. We will present results of the multiple-trait approach in a future communication.

## Conclusion

Combining GP with Monte Carlo simulation can serve as a practical means of detecting superior parents for crop pre-breeding programs. Different strategies can be implemented to identify a set of superior parental lines from a candidate population. The strategy that considers only GEBV will have a higher starting average GEBV among selected parental lines, but it may lead to a less genetic gain. On the other hand, strategies that consider genomic diversity only can retain greater genomic diversity but cannot simultaneously have a favorable starting GEBV average, and therefore may not produce RILs with satisfactory performance. Strategies that consider both GEBV and genomic diversity balance the starting GEBV average and genomic diversity among parental lines; these strategies show satisfactory genetic gain and produce high-performing RILs.

## Supporting information

S1 File R functions. (DOCX)

## Abbreviations

BLUP: best linear unbiased predictor
BRSW: brown rice seed width
FPP: florets per panicle
FT: flowering time
FTAA: flowering time at Arkansas
FTAF: flowering time at Faridpur
GBLUP: genomic best linear unbiased predicto
GD-O: algorithms considering genomic diversity only
GEBV: genomic estimated breeding value
GEBV-GD: algorithms considering both GEBV and genomic diversity
GEBV-O: algorithms considering GEBV only
PH: plant height
PNPP: panicle number per plant
RIL: recombinant inbred line
SNP: single nucleotide polymorphism
YLD: grain yield

## References

[1] Bernardo R. Parental selection, number of breeding populations, and size of each population in inbred development. Theor Appl Genet. 2003;107: 1252–1256. 10.1007/s00122-003-1375-0 12928779

[2] Witcombe JR, Gyawali S, Subedi M, Virk DS, Joshi KD. Plant breeding can be made more efficient by having fewer, better crosses. BMC Plant Biol. 2013;13: 22 10.1186/1471-2229-13-22 23391262 PMC3621587

[3] Hayes BJ, Bowman PJ, Chamberlain AJ, Goddard ME. Genomic selection in dairy cattle: Progress and challenges. J Dairy Sci. 2009;92: 433–443. 10.3168/jds.2008-1646 19164653

[4] Massman JM, Jung HJG, Bernardo R. Genomewide selection versus marker-assisted recurrent selection to improve grain yield and stover-quality traits for cellulosic ethanol in maize. Crop Sci. 2013; 53: 58–66.

[5] Asoro FG, Newell MA, Beavis WD, Scott MP, Jannink JL. Accuracy and training population design for genomic selection on quantitative traits in elite North American oats. Plant Genome 2011;4: 132–144.

[6] Heffner EL, Jannink JL, Sorrells ME. Genomic selection accuracy using multifamily prediction models in wheat breeding program. Plant Genome. 2011;4: 65–75.

[7] Lorenz AJ, Smith KP, Jannink JL. Potential and optimization of genomic selection for *Fusarium* head blight resistance in six-row barley. Crop Sci. 2012;52: 1609–1621.

[8] Spindel J, Begum H, Akdemir D, Virk P, Collard B, Redona E, et al Genomic selection and association mapping in rice (*Oryza sativa*): Effect of trait genetic architecture, training population composition, marker number and statistical model on accuracy of rice genomic selection in elite, tropical rice breeding lines. PLOS Genetics. 2015;11: e1005350 10.1371/journal.pgen.1005350 25689273 PMC4334555

[9] Meuwissen THE, Hayes BJ, Goddard ME. Prediction of total genetic value using genome-wide dense marker maps. Genetics. 2001;157: 1819–1829. 11290733 10.1093/genetics/157.4.1819 PMC1461589

[10] Piepho HP. Ridge regression and extensions for genome-wide selection in maize. Crop Sci. 2009;49: 1165–1176.

[11] Habier D, Fernando RL, Dekkers JCM. The impact of genetic relationship information on genome-assisted breeding values. Genetics 2007;177: 2389–2397. 10.1534/genetics.107.081190 18073436 PMC2219482

[12] VanRaden PM. Efficient methods to compute genomic predictions. J Dairy Sci. 2008;91: 4414–4423. 10.3168/jds.2007-0980 18946147

[13] Gaynor RC, Gorjanc G, Bentley AR, Ober ES, Howell P, Jackson R, et al A two-part strategy for using genomic selection to develop inbred lines. Crop Sci. 2017;57: 2372–2386.

[14] Yao J, Zhao D, Chen X, Zhang Y, Wang J. Use of genomic selection and breeding simulation in cross prediction for improvement of yield and quality in wheat (*Triticum aestivum* L.). Crop J. 2018;6: 353–365.

[15] Vanavermaete D, Fostier J, Maenhout S, De Baets B. Preservation of genetic variation in a breeding population for long-term genetic gain. G3: Genes|Genomes|Genetics 2020; 10.1534/g3.120.401354 32513654 PMC7407475

[16] Zhao K, Tung CW, Eizenga GC, Wright MH, Ali ML, Price AH, et al Genome-wide association mapping reveals a rich genetic architecture of complex traits in *Oryza sativa*. Nat Commun. 2011;2: 467 10.1038/ncomms1467 21915109 PMC3195253

[17] Youens-Clark K, Buckler E, Casstevens T, Chen C, DeClerck G, Derwent P, et al Gramene database in 2010: updates and extensions. Nucleic Acid Res. 2011;39: D1085–D1094. 10.1093/nar/gkq1148 21076153 PMC3013721

[18] Haldane J.B.S. 1919 The combination of linkage values and the calculation of distance between the loci for linked factors. Genetics 8: 299–309.

[19] Henderson CR. Applications of linear models in animal breeding. Univ. of Guelph, Guelph, Ontario; 1984.

[20] Covarrubias-Pazaran G. Genome-assisted prediction of quantitative traits using the R package sommer. PLOS One. 2016;11: e0156744 10.1371/journal.pone.0156744 27271781 PMC4894563

[21] Atkison AC, Donev AN. Optimum experimental designs. New York: Oxford University Press; 1992.

[22] Ou JH, Liao CT. Training set determination for genomic selection. Theor Appl Genet. 2019;132: 2781–2792. 10.1007/s00122-019-03387-0 31267147

[23] Rutkoski JE. A practical guide to genetic gain. Adv Agron. 2019;157: 217–249.

[24] Jia Y, Jannink JL. Multiple-trait genomic selection methods increase genetic value prediction accuracy. Genetics. 2012;192: 1513–1522. 10.1534/genetics.112.144246 23086217 PMC3512156

[25] Hayashi T, Iwata H. A Bayesian method and its variational approximation for prediction of genomic breeding values in multiple traits. BMC Bioinformatics. 2013;14: 1–14. 10.1186/1471-2105-14-1 23363272 PMC3574034

[26] Guo G, Zhao F, Wang Y, Zhang Y, Du L, Su G. Comparison of single-trait and multiple-trait genomic prediction models. BMC Genet. 2014;15: 30 10.1186/1471-2156-15-30 24593261 PMC3975852

