# Supplementary material for: Identification of superior parental lines for biparental crossing via genomic prediction
Source: PLoS One. 2020 Dec 3;15(12):e0243159. doi: 10.1371/journal.pone.0243159 (PMC7714229; doi:10.1371/journal.pone.0243159)
Supplement: S1 File — (DOCX) [file pone.0243159.s001.docx]

**R FUNCTIONS**

##### Function 1: Search a subset of individuals from a candidate set such that the ##### chosen individuals attain the maximal D-score, e.g. the GD-O and ##### GEBV-GD strategies.
# Usage:
# GA.Dscore(K,size,keep,console=F)
# Arguments:
# K: the genomic relationship matrix for the candidate set, e.g. the top 30, 50, 100 # individuals with the highest GEBVs.
# size: the number of individuals to be selected >=4.
# keep: the vector contains the individuals that will be retained before the search, e.g. the # GEBV-GD strategy. Input the ID numbers of the retained individuals. The default is # the empty set for the GD-O strategy.
# console: the iteration process will be displayed in the R console if console=T.
# Output:
# subset: the subset with the maximal D-score.
# D.score: the D-score for the selected subset.
###########################################################################

GA.Dscore=function(K,size,keep=c(),console=F){

 n0=size # the number of chromosomes in the GA algorithm
 if(size>20){n0=20}
 mut=3 # the number of mutations in the GA Algorithm
 if(size<=5){mut=1}
 cri=10000 # the stopping criterion, the algorithm will STOP if the best solution doesn't
 # improve till cri iterations

 p0=1:nrow(K)
 p=p0
 if(length(keep)>0){p=p0[-keep]}

 ans=matrix(0,n0,size)
 for(i in 1:n0){
 ans[i,]=c(keep,sort(sample(p,size-length(keep),replace = F)))
 }
 time=1
 max=-10^10
 improve=10^10
 improvetime=0
 while(time<100000 & improvetime<cri){
 d=c()
 for(i in 1:n0){
 d[i]=det(K[ans[i,],ans[i,]])/(2^size)
 }
 ans=cbind(ans,d)
 ans=ans[order(ans[,(size+1)]),]
 improve=ans[n0,(size+1)]-max
 if(abs(improve)<10^-5){improvetime=improvetime+1
 }else{improvetime=0}
 max=ans[n0,(size+1)]
 result=ans[n0,]

 # selection step
 select1=floor(9*n0/10)
 select2=max-ans[1:select1,(size+1)]
 if(0%in%select2){select2=rep(1,select1)}
 select2=select2/sum(select2)
 select3=sample(1:select1,2,prob=select2)
 ans=ans[-select3,-(size+1)]
 ans.c=ans
 if(length(keep)>0){ans.c=ans[,-(1:length(keep))]}
 if(class(ans.c)[1]!="matrix"){ans.c=matrix(ans.c,length(ans.c),1)}

 # crossover step
 cross1=sample(p,1)
 cross2=sample(1:(n0-2),2,replace = F)
 cross3=ans.c[cross2[1],ans.c[cross2[1],]<cross1]
 cross4=ans.c[cross2[2],!ans.c[cross2[2],]<cross1]
 cross5=c(cross3,cross4)
 if(length(cross5)<(size-length(keep))){
 cross6=sample(p[!p%in%cross5],(size-length(cross5)-length(keep)))
 cross5=sort(c(cross5,cross6))
 } else if (length(cross5)>(size-length(keep))){
 cross5=sort(sample(cross5,(size-length(keep))))
 }
 ans.c=rbind(cross5,ans.c)

 # mutation step
 for(i in 1:(n0-1)){
 mutation1=ans.c[i,]
 mutation2=sample(1:(size-length(keep)),mut,replace = F)
 mutation1=mutation1[-mutation2]
 mutation3=sample(p[!p%in%mutation1],mut)
 mutation4=sort(c(mutation1,mutation3))
 if(i<(n0-1)){ans.c[i,]=mutation4
 }else{ans.c=rbind(ans.c,mutation4)}
 }
 ans=ans.c
 if(length(keep)>0){
 ans=cbind(matrix(keep,nrow(ans.c),length(keep),byrow=T),ans.c)}

 time=time+1
 if(console==T){
 if(time%%200==0 | improvetime==cri){
 print(as.character(c(time,max,improve,improvetime)))}
 }
 }
 D.score=result[(size+1)]
 subset=result[1:size]
 return(list(subset=subset,D.score=D.score,time=time))
}

##### Function 2: Generate the genotype of a gamete by self-pollination.
# Usage:
# simu.gamete(marker)
# Arguments:
# marker: a matrix of order p by 4, where p is the number of markers. The 1st column # contains the chromosome numbers; the 2nd column the marker positions of # linkage map (the unit must be cM); the 3rd and 4th columns the bi-allelic # genotypes for the markers, where 1 indicates the major allele, and -1 the minor # allele.
# Output:
# This function directly returns the genotype of a gamete. A user can redo the function to # generate the required genotypes of gametes. Then, two genotypes are paired to produce # the genotype of a progeny.
###########################################################################

simu.gamete=function(marker){
 marker=marker[order(marker[,1],marker[,2]),]
 distance=marker[,2]
 distance.c = distance[-1]-distance[-length(distance)]
 distance.r = (1-exp(-2*distance.c/100))/2
 distance.r[distance.r<0] = 0.5

 index = sample(3:4,1)
 marker1 = marker[,index]
 marker2 = marker[,(7-index)]

 marker.new = marker1[1]
 for(i in 2:nrow(marker)){
 x = runif(1)
 if(x < distance.r[i-1]){
 marker.new[i] = marker2[i]
 D = marker1
 marker1 = marker2
 marker2 = D
 } else { marker.new[i] = marker1[i] }
 }

 return(marker.new)
}
