# Supplementary material for: Identification of superior parental lines for biparental crossing via genomic prediction
Source: PLoS One. 2020 Dec 3;15(12):e0243159. doi: 10.1371/journal.pone.0243159 (PMC7714229; doi:10.1371/journal.pone.0243159)
Supplement: Attachment — Submitted filename: Response to Reviewers.docx [file pone.0243159.s002.docx]

**Response to Reviewer#1**

Reviewer #1: The strategy for selecting better parental line through genomic prediction (GP) is an excellent idea. This will obviously help the breeder to select the best parents efficiently for getting higher genetic gain. The authors tried to explore GP to select best parental lines for bi-parental crossing based on genomic estimated breeding value (GEBV) couple with genomic diversity (GD) using third party data and simulation study using computer. However, I do have several issue regarding this study and manuscript (MS). I would recommend PlosOne to accept this MS with major revision. Please see below my concern and comments.

Thank you very much for your constructive comments. The following is a point-by-point response.

1. Authors compared the performance of seven strategies for selecting better parental lines by using the actual average value of traits of top 10 lines. They didn’t do any statistical analysis for comparing the significance of the differences. They need to do some kind of statically analysis for any kind of comparing results in this MS.

We have added multiple comparisons by the least significance difference (LSD) test in this revision. The LSD test was performed on the GEBV averages of the best F_10_ RILs, and the genetic gains. We also added the results and discussion about the LSD test.

2. This is a simulation study. In reality, they may not perform similarly since environment will play a big role on the performance of the progeny during the growing cycle of each of the generation up to F10. It would be worthy, if authors can include some kinds of validation work from the real situation.

We have addressed this important point in the DISCUSSION section. Please see the 5^th^ paragraph of the section in this revision.

3. Authors wrote in the line# 320-23, “Apparently, the numbers of accessions fixed in the proposed strategies seem to be a little arbitrary, such as those of selecting 10 parental lines, retaining the top 2 accessions, and searching 10 or another 8 accessions from the three candidate sets composed of the top 30, 50 and 100 accessions, respectively.” My question is why they select it arbitrary instead selecting top based on actual trait value or doing some statistical analysis. Another concern, how they select the top lines for different traits as they could be different for different traits.

We didn’t select the accessions based on their actual trait values, mainly because there are only a fractional of accessions with the actual trait values. The main objective of genomic selection is to predict GEBVs for all possible accessions from the genomic prediction model using the phenotypic data of a training population. In this study, we focus on the single-trait selection, so we may select different top parential lines for different traits. In this revision, we have discussed some potential extensions of the current study to the multiple-trait selection. We will present results of the multiple-trait approach in a future communication. Please see the last paragraph of the DISCUSSION section.

4. They need to rewrite the whole discussion part since many of the statements are redundant with elsewhere especially results section in this MS. They need to include more supporting literature of their outcome. Also they did not explain well of their interesting results.

We have removed the reductant statements, and rewritten most parts of the DISSCUSSION section in this revision. As suggested, we have included some supporting literature for the results.

5. Authors wrote, “An R function for performing the proposed procedure of selecting parental lines is available from the authors upon request”. I would request to authors to include those as supplementary materials for the convenient to readers.

We have put the R function in the supplementary materials.

6. Abstract need to rewrite since more than half contains is background. Only four lines (~15%) results and implication. They need to write more results and outcome from this study.

We have written the ABSTRACT.

7. Some of the sentences are totally identical in the more than one sections.

We have modified the identical sentences in this revision.

8. They need to define first the abbreviation before use in the main body other than abstract.

Done.

9. Authors referred one publication in the MS (line# 58) for those who will interest to learn more. I believe that it is unnecessary.

The citation has been removed in this revision.

10. In the dataset 1, authors used only one third of the available SNPs. My question is how did they select the SNPs and what was the distribution of SNPs in the genome. Please put some numbers.

We have added more details regarding the selection of the SNPs in this revision.

11. Authors wrote that “we then imputed a missing SNP marker from its corresponding major homozygous alleles.” I have no idea how could they imputed a missing marker using alleles data.
We have clarified the sentence, and added more explanations on the coding and imputation of SNPs.

12. Why and how did they reduce the number of progeny (328 from 363) and SNPs (10,772 out of 73, 147) in the dataset 2.

We have explained why we had the reduced number of the individuals, and why we only used such subset of SNPs in this revision.

13. What is the “e” stand for in the equation that measured the recombination rate?

We have added the definition of “e” in this revision.

14. The GEBV average of F10 RILs for yield was the highest in the GD-0-100. However, GEBV for all other yield components were lowest. How is it possible? What are the possible explanation? Please include in the discussion.

Done. Please see the 3^rd^ paragraph of the final section.

15. Authors wrote that “the GEBV averages of the best selected parental lines by the strategies can be ranked as GEBV-O = GEBV-GD-30 = GEBV-GD-50 = GEBV-GD-100 > GD-O-30 > GD-O-50 > GD-O-100 in decreasing desirability”. Results of the corresponding figures 2 and 3 do not support this statement for the all traits.

We have fixed the inequality to match the figures.

16. English language and grammar need to improve. Some references do not have required information such as page number.

We have had a professional editing service for this revised manuscript.

17. In table 2, ranking for PH is not correct.

We have fixed it.
